# Disruption of Dnmt1/PCNA/UHRF1 Interactions Promotes Tumorigenesis from Human and Mice Glial Cells

**DOI:** 10.1371/journal.pone.0011333

**Published:** 2010-06-29

**Authors:** Eric Hervouet, Lisenn Lalier, Emilie Debien, Mathilde Cheray, Audrey Geairon, Hélène Rogniaux, Delphine Loussouarn, Stéphane A. Martin, François M. Vallette, Pierre-François Cartron

**Affiliations:** 1 Centre de Recherche en Cancérologie Nantes-Angers, INSERM, U892, Equipe Aspect mécanistiques et physiopathologiques de l'activité des proteins de la famille de Bcl-2, Equipe labellisée Ligue Nationale Contre le Cancer, Nantes, France; 2 Université de Nantes, Faculté de Médecine, Département de Recherche en Cancérologie, IFR26, Nantes, France; 3 INRA UR1268 Biopolymère Interactions Assemblages, Plate-Forme BIBS, Nantes, France; 4 Service d'Anatomie Pathologique, HGRL, CHU Nantes-Hopital G et R Laennec, Nantes, France; 5 Service de neurochirurgie, CHU Nantes-Hopital G et R Laennec, Nantes, France; University of Hong Kong, Hong Kong

## Abstract

Global DNA hypomethylation is a hallmark of cancer cells, but its molecular mechanisms have not been elucidated. Here, we show that the disruption of Dnmt1/PCNA/UHRF1 interactions promotes a global DNA hypomethylation in human gliomas. We then demonstrate that the Dnmt1 phosphorylations by Akt and/or PKC abrogate the interactions of Dnmt1 with PCNA and UHRF1 in cellular and acelluar studies including mass spectrometric analyses and the use of primary cultured patient-derived glioma. By using methylated DNA immunoprecipitation, methylation and CGH arrays, we show that global DNA hypomethylation is associated with genes hypomethylation, hypomethylation of DNA repeat element and chromosomal instability. Our results reveal that the disruption of Dnmt1/PCNA/UHRF1 interactions acts as an oncogenic event and that one of its signatures (i.e. the low level of mMTase activity) is a molecular biomarker associated with a poor prognosis in GBM patients. We identify the genetic and epigenetic alterations which collectively promote the acquisition of tumor/glioma traits by human astrocytes and glial progenitor cells as that promoting high proliferation and apoptosis evasion.

## Introduction

The low level of DNA methylation in tumors compared to the level of DNA methylation in their normal-tissue counterparts or global DNA hypomethylation was one of the first epigenetic alterations to be found in human cancer [1], [2]. While the contribution of genome hypomethylation in cancer development and progression is explained by several mechanisms: chromosomal instability, loss of imprinting, and reactivation of transposable elements [3], [4], the molecular causes of genome hypomethylation remain unclear. Indeed, despite the central roles of the DNA methyltransferases (Dnmts) in the establishment and maintenance of the DNA methylation, no clear consensus appears between the reduction of the Dnmts expression and the genome hypomethylation in human cancers [5]. Nevertheless, the cancer-associated genome hypomethylation could be explained by the disruption of interactions existing between Dnmts and the DNA replication and DNA repair proteins because these interactions play a crucial role in the DNA methylation in mammalian cells [6], [7], [8]. We here demonstrate that the disruption of the Dnmt1/PCNA/UHRF1 interactions act as oncogenic event promoting the acquisition by human astrocytes and glial progenitor cells of hallmarks of cancer such as high proliferation and apoptosis evasion in a context of genome and gene-specific hypomethylation and chromosomal instability.

## Results

### In glioma, the decrease of the mMTase activity is associated with the degree of DNA hypomethylation and confers poor prognosis of survival

**Figure 1:**
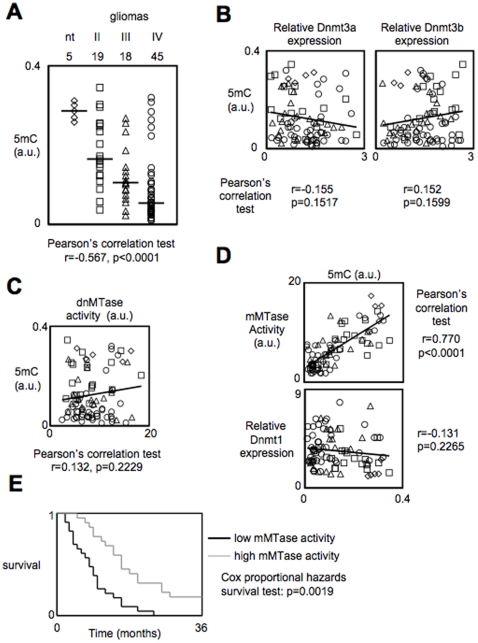
The decrease of maintenance methyltransferase activity (mMTase) is correlated with the genome hypomethylation occurring during gliomagenesis, and confers poor prognosis in glioma patients. (**A**) Correlation between the 5-methylcytosine number (5 mC) and tumor grade in a collection of 82 glioma and 5 non-tumor brain samples (nt). 5 mC was estimated by using the Methylamp Global DNA Methylation Quantification kit (Epigentek-Euromedex, France). Dotted lines represent the median of each parameter. (**B**) Correlation study between the 5-methylcytosine number (5 mC) and level expression of Dnmt3a (left) and Dnmt3b (right). (**C**) Correlation study between the 5-methylcytosine number (5 mC) and the *de novo* methyltransferase (dnMTase) calculated by using unmethylated DNA substrates in DMB assays as previously described [32]. (**D**) Correlation study between the 5-methylcytosine number (5 mC) and the maintenance methyltransferase (mMTase) calculated by using hemi-methylated DNA substrates in DMB assays (Top). Correlation study between relative Dnmt1 expression and the 5-methylcytosine number (5 mC) (bottom). (**E**) Kaplan-Meier estimates time of survival between patients suffering from glioma presenting a high level of mMTase activity (grey line) and those whose tumors harbored a low level of mMTase activity (black line). In this figure, ⋄ represents 5 non-tumor brain samples, □ represents grade II astrocytomas/oligodendroglioma, ▵ represents grade III astrocytomas/oligodendroglioma, ○ represents grade IV astrocytomas/GBM.

The global DNA methylation status of glioma was assessed by measuring the number of 5-methylcytosine (5 mC) in a collection of 82 surgical resections of glioma and in 5 non-pathological brain biopsies (**Figure 1A**). ELISA results indicate that the 5 mC number decreases when the glioma grade increases indicating that the genome hypomethylation characterizes the initiation and/or the development of gliomagenesis (Pearson's correlation test, r = −0.537, p<0.0001). To identify a molecular cause of global DNA hypomethylation, we initially searched whether the decrease of 5 mC number occurring during gliomagenesis is inversely correlated with the expression level of Dnmt3a and Dnmt3b or with their methyltransferase (MTase) activity i.e. with the *de novo* MTase activity. No significant correlation was reveled by statistical analysis of these parameters (r = −0.155, p = 0.1517, r = 0.152, p = 0.1599 and r = 0.132, p = 0.2229, respectively) (**Figures 1B and 1C**).

The Dnmt1 being the predominant maintenance methyltransferase enzyme, we next assessed its expression and its activity (i.e. the maintenance MTase (mMTase) activity) in glioma biopsies in order to determine whether the alteration of these parameters could explain the global DNA hypomethylation seen in glioma. These analyses indicated that the decrease of 5 mC number occurring during gliomagenesis is correlated with the decrease of mMTase activity but not with the variations of the expression level of the Dnmt1 (r = 0.770, p<0.0001 and r = −0.131, p = 0.2265, respectively) (**Figure 1D**).

We then implemented this observation by analyzing whether the level of mMTase activity could be used as an alternative prognostic factor in a group of 45 GBM patients for which we obtained a well-documented medical history (**Supplemental data S1**). Based on the mMTase activity levels, the 45 patients were divided into two subgroups. 23 patients whose glioma presented a low level of mMTase activity (i.e. equal to or lower than the median value of mMTase activities) were included in group#1, while 22 patients whose tumors harbored a high level of mMTase activity (i.e. higher than the median value of mMTase activities) composed group#2. Survival curves were estimated by the Kaplan-Meier method and compared with the Cox proportional hazards survival regression test (**Figure 1E**). Thus, we observed a significant difference in survival time between patients who had a high level of mMTase and those who did not (p = 0.0019). These results indicate that the level of mMTase activity could be used as a prognostic factor for survival. Moreover, the identification of molecular mechanisms implicated into the global DNA hypomethylation via the decrease of mMTase activity would open new and rational biomarkers for patient selection in anti-glioma therapy and would identify new therapeutic targets against glioma.

### The low level of the Dnmt1/PCNA/UHRF1 interactions is a molecular hallmark associated with the low degree of global DNA hypomethylation in glial/glioma cells

**Figure 2:**
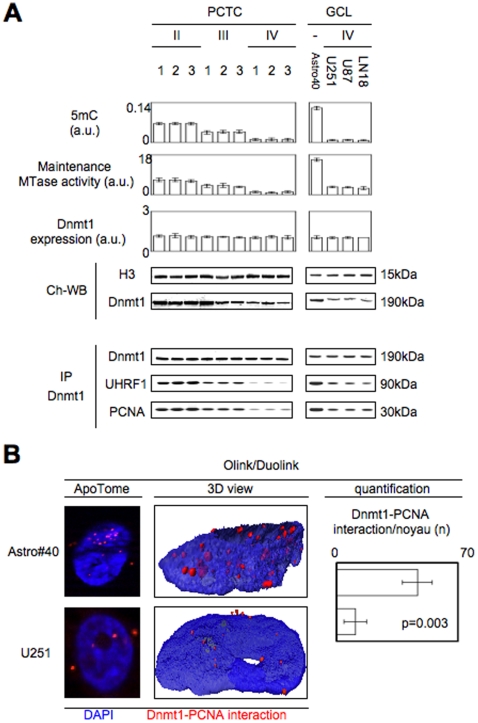
Disruption of the Dnmt1/PCNA and Dnmt1/UHRF1 interactions in gliomagenesis. (**A**) 5-methylcytosine (5 mC) number, maintenance methyltransferase (mMTase) activity, Dnmt1 expression, Dnmt1 recruitment on DNA and Dnmt1/PCNA/UHRF1 interaction in a panel of PCTC issued from the different grade of glioma. 5 mC was estimated by using the Methylamp Global DNA Methylation Quantification kit (Epigentek-Euromedex, France). mMTase activity and Dnmt1 expression was measured from nuclear extract obtained by using the EpiQuik Nuclear Extraction Kit I (Euromedex, France). Immunoprecipitations are performed by using the Catch and Release® v2.0 Reversible Immunoprecipitation System (Millipore, France). (**B**) Use of proximity ligation *in situ* assay (P-LISA) to monitor the disruption of the Dnmt1-PCNA interactions. Nucleus/DNA are in blue and Dnmt1-PCNA interaction in red. Quantification was performed from the analysis of 100 cells of three independent experiments.

To identify the molecular mechanism governing the decrease of mMTase and conferring the increase of the degree of global DNA hypomethylation characterizing the human gliomagenesis, we decided to screen the level of 5 mC, the mMTase activity and the Dnmt1 expression in a panel of glial/glioma cells including nine primary cultured tumor cells (PCTC) obtained from glioma of different grades (3 PCTC per glioma grade), and four glial cell lines (GCL) in which Astro#40 represents non tumoral human astrocytes and U251, U87 and LN18 cells represent tumor human glioma cell lines. Consistently with what we reported about the human biopsies, ELISA, flow cytometry and DMB assay indicated that the 5 mC number and the mMTase activity decreased during gliomagenesis while the Dnmt1 expression remained unchanged (**Figure 2A** **and Supplemental data S2**). In parallel with these results, western blot realized from chromatin extraction revealed that the decrease of mMTase activity, seen in glioma, is associated with the decrease of the Dnmt1 quantity recruited on DNA (**Figure 2A**).

Literature reporting that PCNA and UHRF1 proteins mediate the epigenetic inheritance in mammalian cells by recruiting Dnmt1, we then hypothesized that the defect of Dnmt1 recruitment on DNA could be due to the disruption of interactions existing between Dnmt1, PCNA and UHRF1 [6], [8]. The Dnmt1-immunoprecipitation indicated that it was the case because the quantity of PCNA and UHRF1 decreased when the tumor grade increased while the quantity of Dnmt1 immunoprecipitated remained unchanged (**Figure 2A** **and Supplemental data S3**). The disruption of the Dnmt1/PCNA interactions is also confirmed by the use of a proximity ligation *in situ* assay (P-LISA) and the significant reduction of the Dnmt1/PCNA interactions in U251 cells (a glioma cell line) compared with Astro#40 cells (a non-tumor glial cell lines) (p = 0.003) (**Figure 2B**).

Collectively, all our data identified the disruption of the Dnmt1/PCNA/UHRF1 interactions as a molecular event associated with the degree of global DNA hypomethylation in glial/glioma cells.

### The phosphorylations of the Dnmt1 at residues preferentially phosphorylated by Akt and PKC is a molecular hallmark associated with the low level of the Dnmt1/PCNA/UHRF1 interactions and with the low degree of global DNA hypomethylation in glial/glioma cells

**Figure 3:**
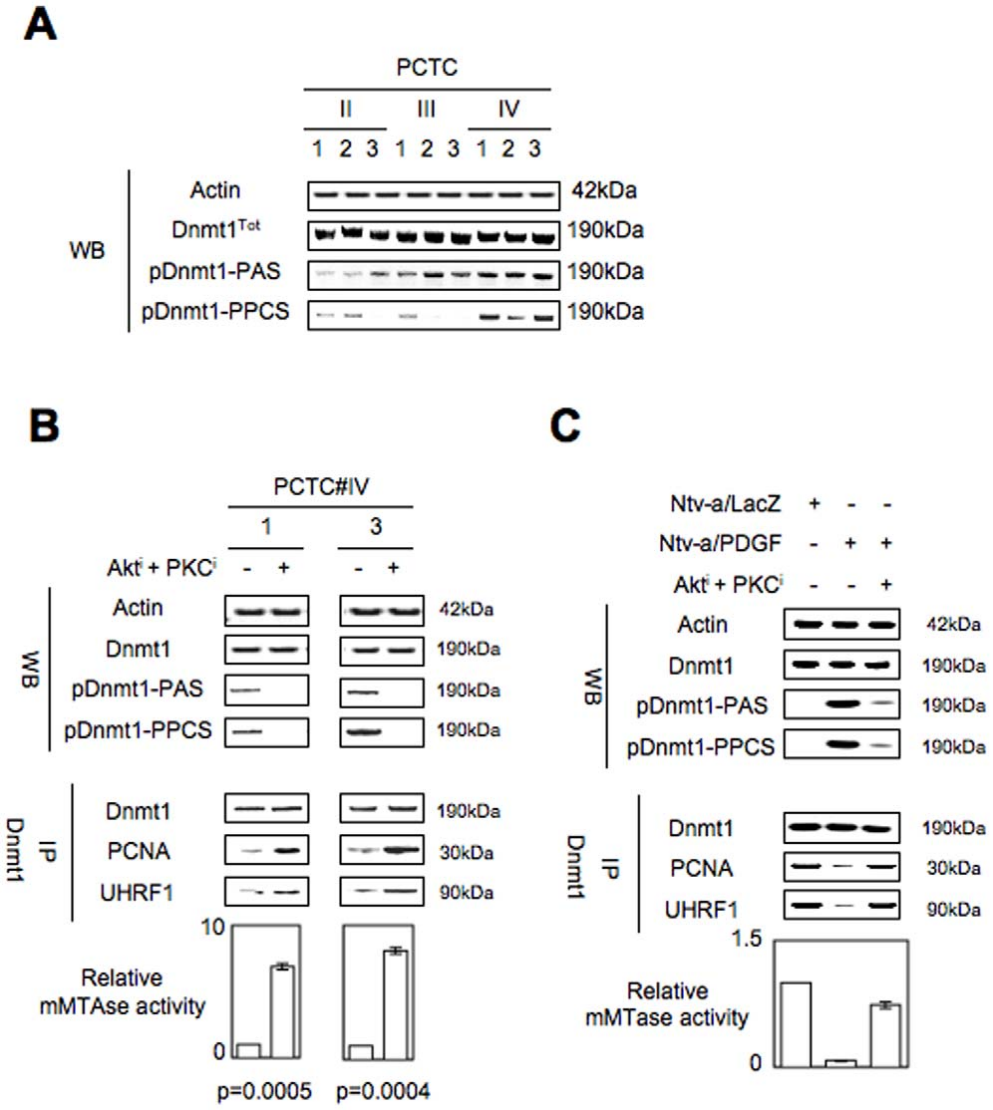
The disruption of the Dnmt1/PCNA/UHRF1 interactions is associated with the phsphorylation of Dnmt1 by Akt and/or PKC. (**A**) Visualization, in primary cultured tumor cells (PCTC) of different grade of glioma, of the phosphorylation level of Dnmt1 by using antibodies recognizing the phospho-(Ser/Thr) Akt substrate (pDnmt1-PAS, Ozyme, Cell Signal#9614, France) and the the phospho-(Ser) PKC substrate (pDnmt1-PPCS, Ozyme, Cell Signal#2261, France). (**B**) Effect of the Akt and PKC inhibition on the phosphorylation level of Dnmt1, the Dnmt1-PCNA-UHRF1 interactions and on the mMTAse activity. (Akt^i^: 0.1 µM Calbiochem#124005, France; PKC^i^ or Go6893, 0.5 µM Calbiochem#124005, France). (**C**) Effect of the constitutive activation of Akt and PKC in Ntv-a/PDGF cells on the phosphorylation level of Dnmt1, the Dnmt1-PCNA-UHRF1 interactions and on the mMTAse activity.

In parallel with these results, we noted that the phosphorylation levels of Dnmt1, at residues preferentially recognized and phosphorylated by Akt and PKC (pDnmt1-PAS and pDnmt1-PPCS, respectively), increased when the tumor grade increased (**Figure 3A**). In other terms, this result indicated that the levels of pDnmt1-PAS and pDnmt1-PPCS are inversely correlated with the level of the Dnmt1-PCNA-UHRF1 interactions. Thus, we wondered whether these phosphorylations of Dnmt1 are hallmarks reflecting the level of the Dnmt1-PCNA-UHRF1 interactions in two cellular systems of study.

By treating the PCTC#IV with Akt and PKC inhibitors, we firstly demonstrated that the loss of pDnmt1-PAS and pDnmt1-PPCS is associated with the increase of the Dnmt1-PCNA-UHRF1 interactions and with the increase of mMTase activity i.e. of the enzymatic activity mainly catalyzed by the Dnmt1 (**Figure 3B**).

Secondly, by promoting the activation of Akt and PKC via the constitutive overexpression of PDGF-B in Ntv-a/RCAS system of gliomagenesis, we induced the presence of pDnmt1-PAS and pDnmt1-PPCS and the strong decrease of the Dnmt1-PCNA-UHRF1 interactions and of the mMTase activity (**Figure 3C**). The link between these three events is also supported by the fact that the treatment of the Ntv-a/PDGF cells with Akt and PKC inhibitors reduced the presence of pDnmt1-PAS and pDnmt1-PPCS and restored the Dnmt1-PCNA-UHRF1 interactions (**Figure 3C**).

Thus, it seems that, among the pleotropic effects of the PDGF signaling, the activation of Akt and PKC is the main effect responsible of the decrease of the mMTase activity. This point is also supported by the fact that the inhibition of the Ras signaling pathway (one other major PDGF-induced signaling pathway) in the Ntv-a/PDGF cells via a farnesylthiosalicylic acid (FTS) (40 µM) treatment, did not affect the mMTase activity (p = 0.8143) (**Supplemental data S4**).

### Identification and impact of the Akt- or PKC-induced phosphorylations of the Dnmt1 on its capacity to interact with PCNA and UHRF1

**Figure 4:**
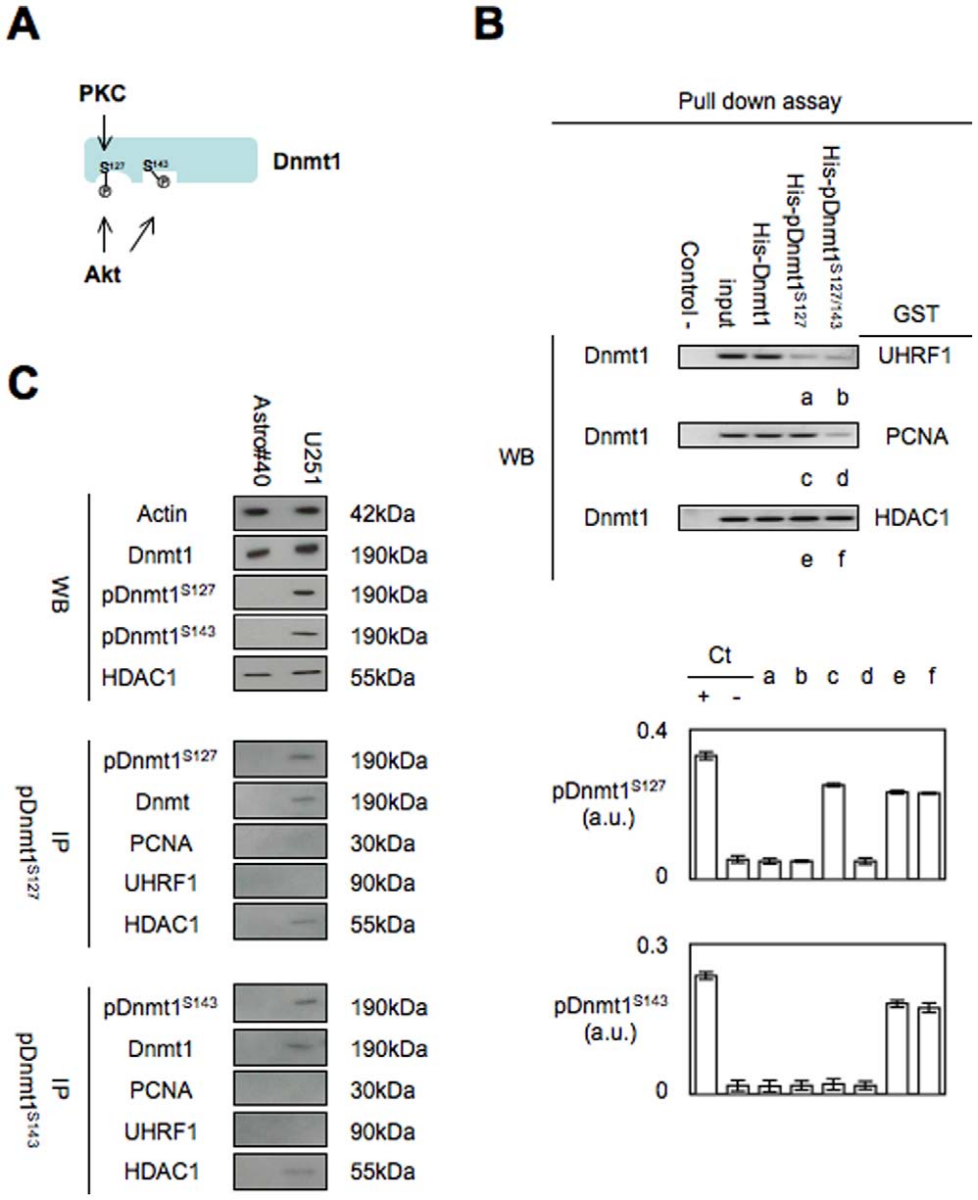
The phsophorylation of the Dnmt1 at S127 and/orS143 decrease the capacity of the protein to interact with PCNA and UHRF1. (**A**) Schematic representation of the Akt and PKC-induced phosphorylation of Dnmt1 according to the results obtained by mass spectrometric approach. (**B**) Effect of the phosphorylation of the recombinant Dnmt1 (Methylation Ltd, Port Orange, Florida) by Akt and PKC on the Dnmt1-PCNA, Dnmt1-UHRF1 and Dnmt1-HDAC1 interactions. Pull-down assays were performed by using the GST/His Tag Protein Interaction Pull-Down Kits (Thermo Scientic, France). Fusion protein purification gel is illustrated in **Supplemental data S5**. The phoshorylation level of the Dnmt1 interacting with GST-PCNA, GST-UHRF1 and GST-HDAC1 was analyzed by ELISA method. Ct+: Dnmt1^R^ phosphorylated by Akt and/or PKC, Ct-: Dnmt1^R^. (**C**) Western blot analyses were realized to monitor the expression level of Dnmt1, pDnmt1^S127^, pDnmt1^S143^ and HDAC1, Actin was used as control. IP experiments were realized by using the Catch and Release® v2.0 Reversible Immunoprecipitation System (Millipore, France) with 4 µg of antibody. Western blot of IP experiments illustrate the results obtained by adding the immunoprecipitate obtained from 3 immunoprecipitations performed with 500 µg of proteins.

To identify the amino acid residue phosphorylated by Akt and PKC, we decided to perform mass spectrometric analysis from recombinant Dnmt1 protein (Dnmt1^R^) phosphorylated by Akt and PKC. Thus, despite the weak presence of the pDnmt1^S127^ into the Dnmt1^R^ purified from baculovirus, mass spectrometric analyses clearly indicated that Akt and PKC phosphorylated the Dnmt1^R^ at residues serine-127/143 and serine-127 respectively (**Figure 4A** **and Supplemental data S6**).

According to these data, we synthesized two antibodies directed against the pDnmt1^S127^ and pDnmt1^S143^ (Proteogenix, France). After validation of these antibodies by using kinase assays, western blot and ELISA methods, we decided to use these the antibodies to determine the impact of the Akt/PKC-induced phosphorylations of Dnmt1 on the Dnmt1/PCNA and Dnmt1/UHRF1 interactions (**Supplemental data S7 and Supplemental data S8**). For this purpose, we realized pull down and immunoprecipitation experiments.

In pull down experiments, we generated the pDnmt1^S127^ and pDnmt1^S127/S143^ proteins by using the recombinant PKC and Akt kinases (**Supplemental data S8**). Thus, we noted that the degree of phosphorylation of Dnmt1 at S127 by PKC decreased the Dnmt1/UHRF1 interactions without affected the Dnmt1/PCNA interactions (**Figure 4B**). The double phosphorylation of Dnmt1 at S127 and S143 by Akt decreased the Dnmt1/UHRF1 and Dnmt1/PCNA interactions. No effect of the Akt/PKC-induced phosphorylations of Dnmt1 was observed about the Dnmt1/HDAC1 interactions. Moreover, an ELISA analysis performed with the pDnmt1^S127^ and pDnmt1^S143^ antibodies, revealed that the Dnmt1 interacting with GST-UHRF1 was unphosphorylated, that the Dnmt1 interacting with GST-PCNA can be phosphorylated at residus S127, and that the Dnmt1 interacting with GST-HDAC1 can be phosphorylated at residus S127 and/or S143 (**Figure 4B**).

By performing western blot and immunoprecipitation experiments from Astro#40 and U251 cells, we not detected the presence of the pDnmt1^S127^ and/or pDnmt1^S143^ in Astro#40 cells, while these two forms of Dnmt1 are present in U251 cells (**Figure 4C**). Despite the weak quantity of Dnmt1 immunoprecipitated by using the pDnmt1^S127^ or pDnmt1^S143^ antibodies, our data clearly indicated that these two forms of Dnmt1 are devoid of interaction with PCNA and UHRF1, but conserved their interactions with HDAC1 (**Figure 4C**).

Thus, these two last results strongly demonstrated that the Akt/PKC phosphorylation of Dnmt1 is a hallmark dictating whether the Dnmt1 interact or not with PCNA and/or UHRF1 interactions. Besides, this results is consistent with the fact that the Akt- and PKC-mediated phosphorylations of Dnmt1 occur in regions included or juxtaposed the interaction domains of Dnmt1 with PCNA or UHRF1 [6], [7], [9]. The use of the pDnmt1^S127^ and pDnmt1^S143^ antibodies also confirmed the fact that the Akt/PKC phosphorylation of Dnmt1 is a hallmark dictating whether the Dnmt1 interact or not with PCNA and/or UHRF1 interactions since we observed a correlation between the level of phosphorylation of Dnmt1 in PCTC used in figures 3A and 3B and the level of the Dnmt1/PCNA/UHRF1 interactions in these cells (**Supplemental data S9**). Thus, all these experiments indicated and reinforced the idea that the increase of the presence of pDnmt1^S127^ and/or pDnmt1^S143^ is performed to the detriment of the presence of the Dnmt1/PCNA/UHRF1.

### pDnmt1^S127/S143^ and/or pDnmt1^S127^ catalyze low mMTase activity in comparison with Dnmt1/PCNA/UHRF1 and is hallmark associated with poor prognosis in glioma

**Figure 5:**
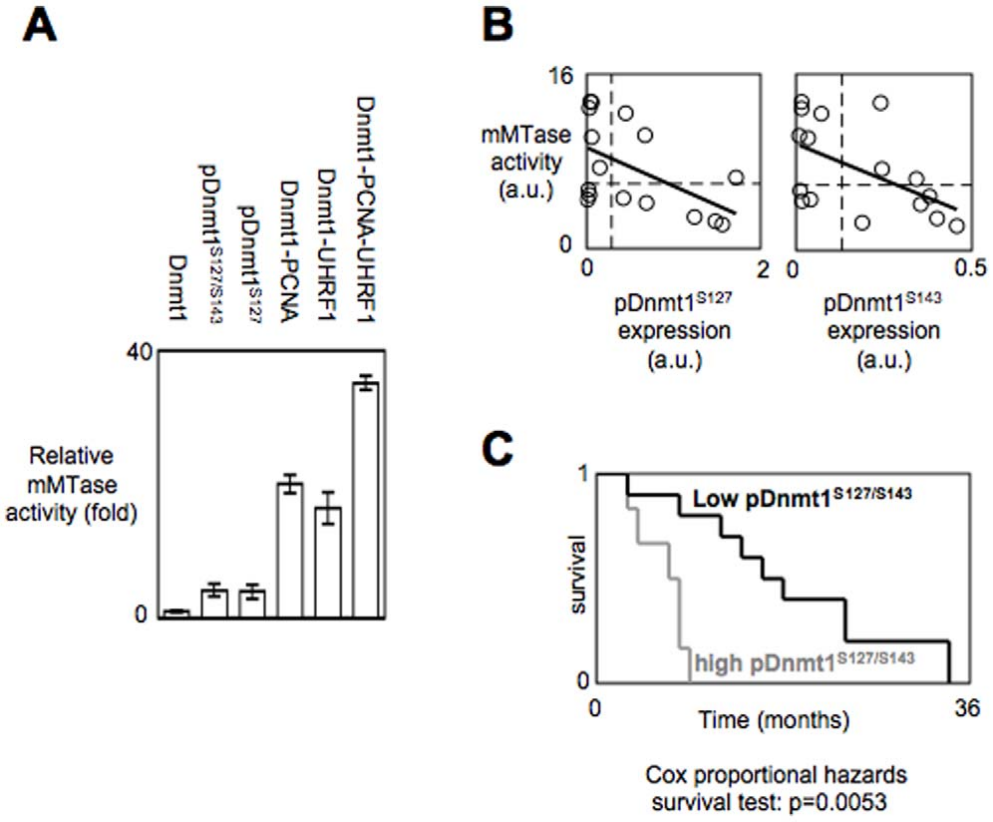
pDnmt1^S127/S143^ and/or pDnmt1^S127^ catalyze low mMTase activity in comparison with Dnmt1/PCNA/UHRF1 and is hallmark associated with poor prognosis in glioma. (**A**) Analysis of the mMTAse activity catalyzed by the Dnmt1, the Akt-mediated phosphorylation of the Dnmt1 (pDnmt1-PAS), the PKC-mediated phosphorylation of the Dnmt1 (pDnmt1-PPCS), in presence of equimolar quantity of PCNA, (Dnmt1-PCNA), or UHRF1 (Dnmt1-UHRF1) or PCNA and UHRF1 (Dnmt1-PCNA-UHRF1). mMTAse activities were assessed by DMB assay according to Yokochi and Robertson (2004). (**B**) Correlation study between the mMTase activity and the expression level of pDnmt1^S127^ and pDnmt1^S143^ harbored by 16 GBM. ○ represents grade IV astrocytomas/GBM. (**C**) Kaplan-Meier estimates time of survival between patients suffering from glioma presenting a high expression level of pDnmt1^S127^ and pDnmt1^S143^ (grey line) and those whose tumors harbored a low expression level of pDnmt1^S127^ and pDnmt1^S143^ (black line).

To determine whether the presence of the pDnmt1^S127^ and/or pDnmt1^S127/S143^ to the detriment of the presence of the Dnmt1/PCNA/UHRF1 is a situation associated with the decrease of the mMTAse activity catalyzed by the Dnmt1, we next measured the mMTase activity catalyzed by the Dnmt1, the pDnmt1^S127/S143^, the pDnmt1^S127^, the Dnmt1 in presence of PCNA (Dnmt1-PCNA), UHRF1 (Dnmt1-UHRF1) or PCNA and UHRF1 (Dnmt1-PCNA-UHRF1). As illustrated by the **figure 5A**, we noted that the phosphorylation of Dnmt1 by Akt or PKC increased the mMTase activity of the Dnmt1 since pDnmt1^S127/S143^ and pDnmt1S^127^ catalyzed 4-fold more incorporation of methyl group ^3^H-radiolabelled than the Dnmt1. Our analysis also indicated that the mMTase activity of pDnmt1^S127/S143^ and pDnmt1S^127^ are inferior to the mMTase activity catalyzed by Dnmt1/PCNA (5-fold), Dnmt1/UHRF1 (4-fold) or Dnmt1/PCNA/UHRF1 (9-fold). Thus, this data underline the existence of a hierarchy of mMTase activity catalyzing by the Dnmt1 in function its phosphorylation status and of its partners of interaction. Besides, this hierarchy is consistent with our previous results.

Our previous data indicating that the low mMTAse activity is a poor prognosis factor, we now wondered whether the expression level of pDnmt1^S127^ and/or pDnmt1^S143^ could be used as a molecular marker of poor prognosis since the presence of these two phsophorylated forms of Dnmt1 is associated with a low mMTAse activity. In 16 GBM expressing similar level of Dnmt1, we noted that the levels of pDnmt1^S127^ and/or pDnmt1^S143^ were inversely correlated with the level of mMTase harbored by the corresponding GBM (Pearson's correlation test, r = −0.535, p = 0.0327; r = −0.531, p = 0.0343) (**Figure 5B**).

Thus, these two results strongly support the idea that the pDnmt1^S127^ and pDnmt1^S127/S143^ is less efficient to catalyze the mMTase activity. In addition to being correlated with a low mMTase activity, Kaplan-Meier method and Cox proportional hazards survival regression analysis also revealed that the presence of the pDnmt1^S127^ and/or pDnmt1^S127/143^ is associated with a poor prognosis factor (p = 0.0053) (**Figure 5C**). Thus, the detection of the pDnmt1^S127^ and/or pDnmt1^S143^ in GBM appears as a promising molecular biomarker that could be used as an alternative predictor of disease outcome.

### The disruption of the Dnmt1/PCNA/UHRF1 interactions promotes the global DNA hypomethylation in astrocytes (Astro#40) and in glial precusor cells (Ntv-a)

**Figure 6:**
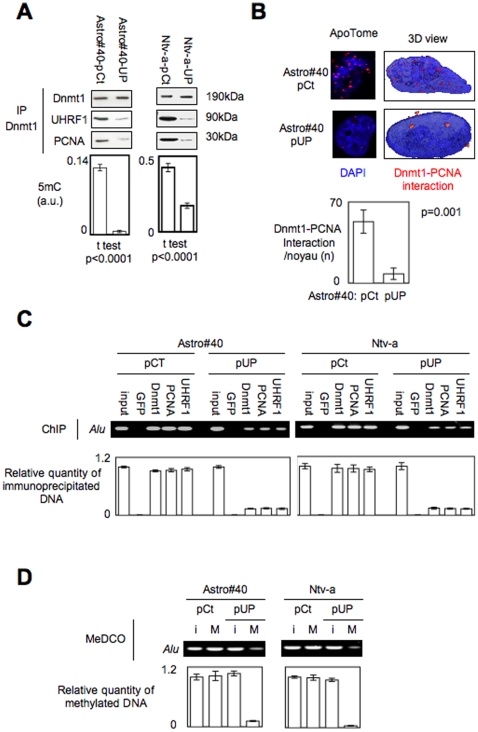
The disruption of the Dnmt1/PCNA/UHRF1 interactions promotes the global DNA hypomethylation in astrocytes (Astro#40) and in glial precusor cells (Ntv-a). (**A**) Monitoring by immunoprecipitation of Dnmt1 and ELISA of the effect of the expression of the UP protein” (a chimera protein composed by the 163–171 amino-acids of PCNA and the 526–614 amino-acids of UHRF1) on the Dnmt1/PCNA/UHRF1 interactions and on the level of 5-methylcytosine (5 mC). (**B**) Use of proximity ligation *in situ* assay (P-LISA) to monitor the “UP”-induced disruption of the Dnmt1-PCNA interactions. Nucleus/DNA are in blue and Dnmt1-PCNA interaction in red. Quantification was performed from the analysis of 100 cells of three independent experiments. (**C**) Impact of the “UP”-induced disruption of the Dnmt1-PCNA-UHRF1 interactions on the co-recruitment of Dnmt1, PCNA and UHRF1 on Alu, a DNA repeat element. Chromatin Immunoprecipitation (ChIP) was performed by using the EZ-ChIP (Millipore, France). For each point, the relative quantity of immunoprecipitated DNA is obtained by using input as reference. (**D**) Impact of the “UP”-induced disruption of the Dnmt1-PCNA-UHRF1 interactions on the methylation status of Alu by coupling the Methylated DNA COllection and PCR amplification (MeDCO) via the use of the MethylCollector Ultra kit (Active Motif, France). (I:input: M:Methylated and collected DNA).

We then asked whether the disruption of these interactions, as a single event, could generate the global DNA hypomethylation in Astro#40 (astrocytes) and/or Ntv-a (glial precursors) cells. For this purpose, we have transfected Astro#40 cells and Ntv-a cells with pUP plasmid, i.e. a plasmid coding amino-acid regions of Dnmt1 (163–173aa) and UHRF1 (596–614aa) interacting with Dnmt1 [6], [7], [9] (**Supplemental data S10**). Dnmt1-immunoprecipitation and P-LISA strongly demonstrated that the Dnmt1-PCNA-UHRF1 interactions were disrupted in Astro#40/pUP and Ntv-a/pUP cells (**Figures 6A and 6B**). ELISA revealed that the latter cells displayed a lower level of 5 mC than its parental cells (p<0.0001) (**Figure 6A**). By performing Chromatin Immunoprecipitation (ChIP) and Methylated DNA collection (MeDCO), we noted that the transfection of Astro#40 and Ntv-a cells with the pUP plasmid decreased the co-recruitment of Dnmt1, PCNA and UHRF1 on Alu, a DNA repeat element, and reduced its degree of methylation (**Figures 6C and 6D**). Thus, our data clearly identify that the forced disruption of the Dnmt1/PCNA/UHRF1 interactions as a molecular determinant of global DNA hypomethylation.

### The disruption of the Dnmt1/PCNA/UHRF1 interactions, in astrocytes (Astro#40) and in glial precusor cells (Ntv-a), promotes chromosomal instability and gene-specifc hypomethylation

**Figure 7:**
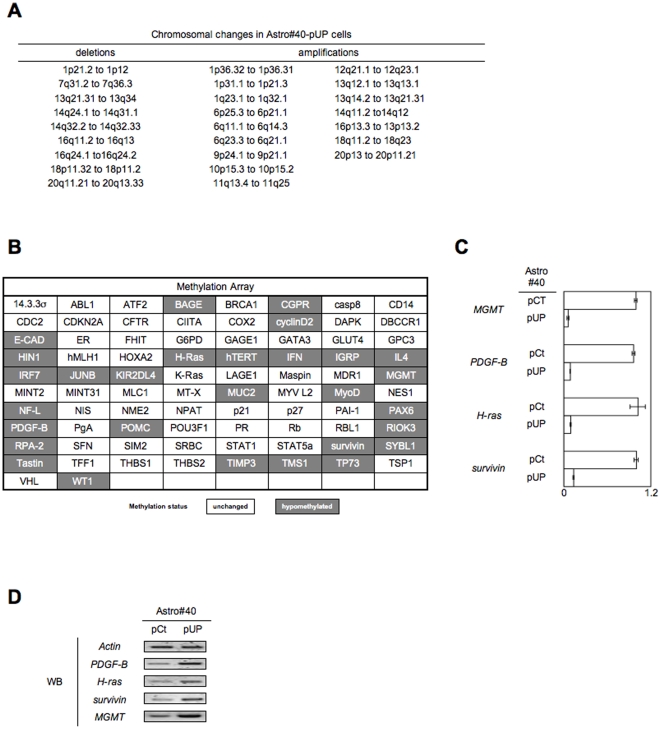
The disruption of the Dnmt1/PCNA/UHRF1 interactions, in astrocytes (Astro#40) and in glial precusor cells (Ntv-a), promotes chromosomal instability and gene-specifc hypomethylation. (**A**) Description of chromosomal changes in Astro#40-UP cells via the realization of CGH array. (**B**) Description of changes in the methylation profil Astro#40-UP cells via the realization of Methylation Array (Ozyme-Panomics, France). (**C**) Validation by Methylated DNA Immunoprecipitation (MeDIP) and PCR amplification (MeDIP) of the hypomethylation of the *MGMT, PDGF-B, H-ras* and *survivin* genes in Astro40#UP cells. (I:input: M:Methylated and collected DNA). For each point, the relative quantity of immunoprecipitated DNA is obtained by using input as reference. (**D**) Expression level of the MGMT, PDGF-B, H-ras and survivin proteins in Astro#40 and Astro40#UP cells via western blot analysis.

Hypomethylation of DNA repeat element being able to induce chromosomal instability, we next performed a comparative genome hybridization array (CGH-array) using DNA from Astro#40/pCt and Atrso#40/pUP cells [10], [11]. Significant changes in chromosomes organization of Astro#40/pUP cells compared to parental cells were observed such as 9 deletions and 16 amplifications of specific chromosomal regions (**Figure 7A**).

We extended our study by investigating the impact of the global DNA hypomethylation on the methylation status of specific genes since DNA hypomethylation has the potentially to activating oncogenes or genes involved in tumorogenesis. The comparison of gene-specific methylation profiles obtained by Promoter Methylation Array (Ozyme, France) revealed that the decrease of 5 mC number in Astro#40/pUP cells as compared with that of Astro#40/pCt cells is accompanied by the hypomethylation of 29 genes (**Figure 7B**). Among the hypomethylated genes, MeDCO analysis validated the fact that, *PDGF-B, H-ras, survivin* or *MGMT* genes were hypomethylated in Astro#40/pUP cells by comparison with the Astro#40 cells (**Figure 7C** **and Supplemental data S11**). Finally, western blot indicated that the hypomethylation of these genes is associated with their overexpression at protein level (**Figure 7D**). Similar results are also obtained by analyzing the Ntv-a/pCt and Ntv-a/pUP cells (**Supplemental data S12**).

Collectively, our data indicated that the disruption of the Dnmt1-PCNA-UHRF1 interactions is a molecular event inducing chromosomal instability and hypomethylation-initiated overexpression of oncogenes such as PDGF or H-ras.

### The disruption of the Dnmt1/PCNA/UHRF1 interactions promotes the tumor transformation of astrocytes (Astro#40) and glial precusor cells (Ntv-a)

**Figure 8:**
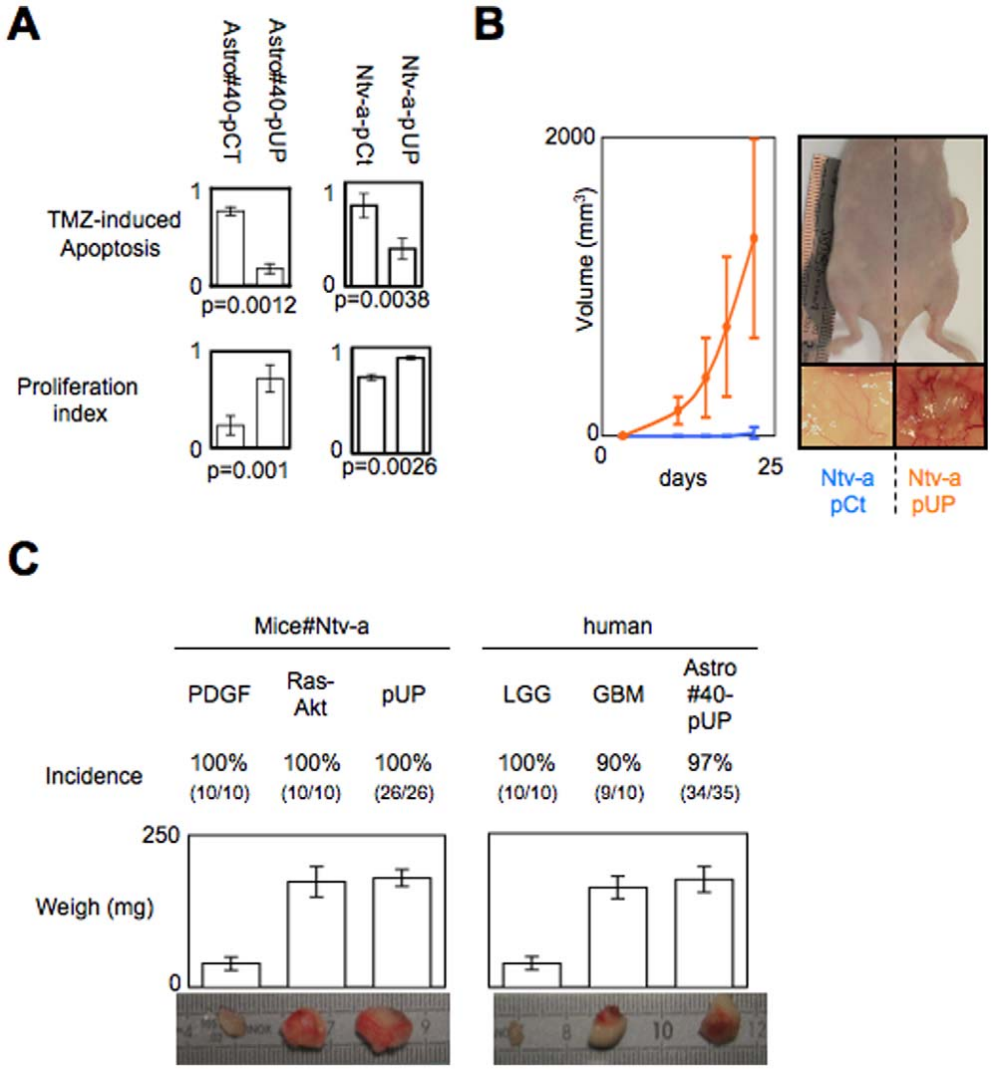
Impact of the “UP”-induced disruption of the Dnmt1/PCNA/UHRF1 interactions on hallmarks of cancer in Astro#40 and Ntv-a cells. (**A**) Comparison of the temozolomide-induced apopto-sensitivity (*via* the measure of DEVDase activity) and the proliferation index between the Astro#40 and Astro#40-UP cells and between the Ntv-a and Ntv-a/UP cells. DEVDase activity was assessed as previously described[33]. Proliferation index was evaluated by quantify the cells number. (**B**) Tumorigenicity test of the Ntv-a and Ntv-a/UP cells. (**C**) Comparison of the tumorogenicity of the Ntv-a/UP cells with the one of the Ntv-a/PDGF and Ntv-a/Ras-Akt cells (left) and of the tumorogenicity of the Astro#40-UP cells with the one of the primary cultured tumor cells (PCTC) obtained from low-grade glioma (LGG) or glioblastoma multiforme (GBM) (right). Pictures are representative of tumors obtained after cells injection.

To determine whether the disruption of the Dnmt1/PCNA/UHRF1 interactions can promote the tumor transformation of Astro#40 and Ntv-a cells, we firstly investigated whether these cells acquired some hallmark of cancer after their transfection by the pUP plasmid. As illustrated by the **figure 8A**, we noted that it was the case since the Ntv-a/pUP and Astro#40/pUP cells are more proliferating and more resistant to temozolomide-induced apoptosis than the Ntv-a/pCT and Astro#40/pCt cells. Secondly, we have tested the tumorogenicity of the Ntv-a/pUP and Astro#40/pUP cells in nude mice. Thus, tumorogenicity assay performed *via* the *s.c.* injection of cells in nude mice showed that the presence of macroscopically visible tumors in 100% (26/26) injections of Ntv-a/UP cells, (**Figure 8B**), while no tumor pushes after injections of Ntv-a cells. Similarly, we noted that the presence of macroscopically visible tumors in 97% (34/35) injections of Astro#40-UP cells (**Figure 8C**). We then compared the growth pattern of the tumors developed from the injection of Ntv-a/UP or Astro#40-UP cells with the one developed from the injection of the Ntv-a/PDGF (grade II glioma), Ntv-a/Ras-Akt (grade IV glioma), PCTC#II, and PCTC#IV. As illustrated by the **figure 8C**, it appears that the growth pattern of tumors developed from the injection of Ntv-a/UP or Astro#40-UP cells are similar to the ones obtained from the injection of cells of grade IV glioma (i.e. Ntv-a/Ras-Akt or PCTC#IV cells). Thus, all these results clearly demonstrate that the disruption of the Dnmt1/PCNA/UHRF1 interactions acts as an oncogenic event inducer of gliomagenesis.

**Figure 9:**
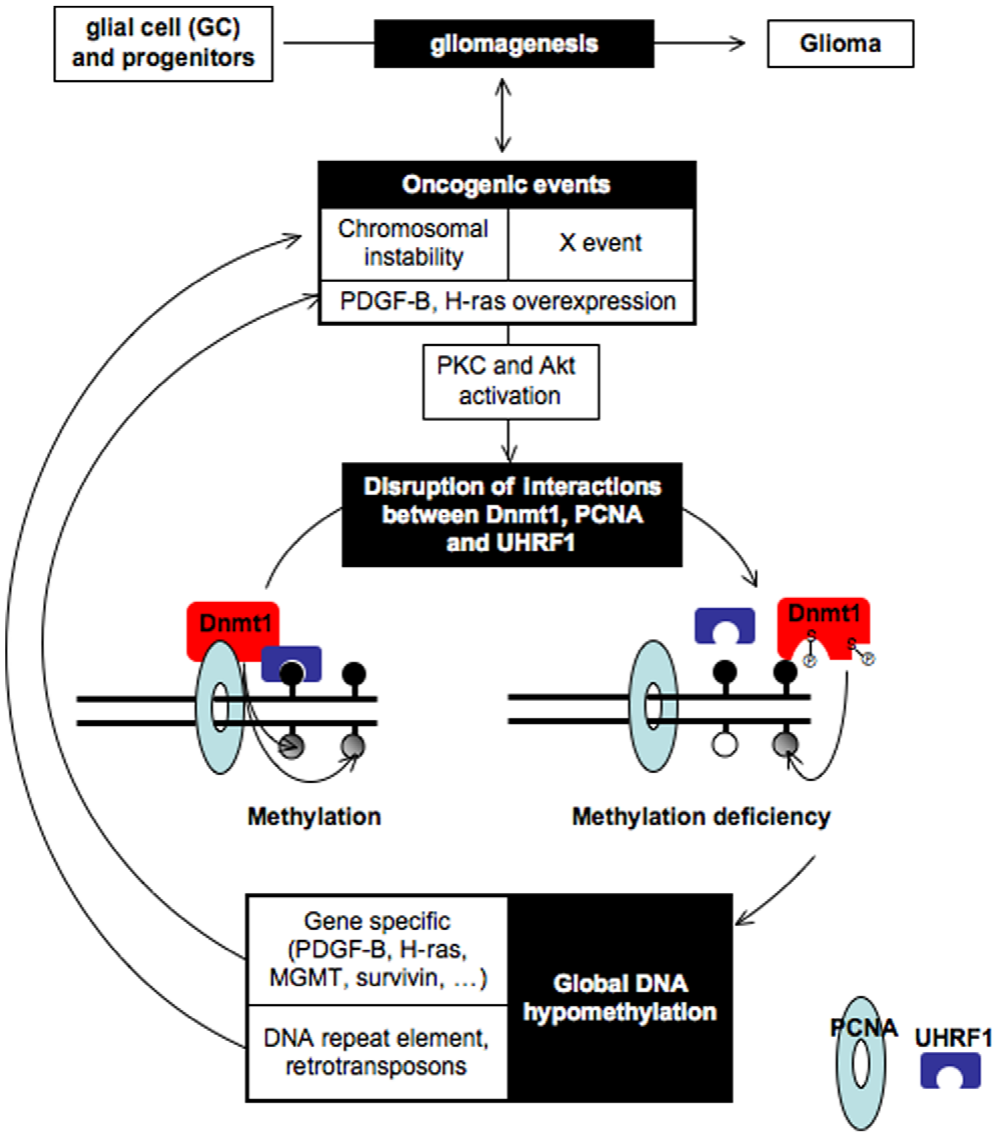
Schematic representation of gliomagenesis induced by the disruption of the Dnmt1-PCNA-UHRF1 interactions.

In summary, our results underline, for the first time, that the disruption of the Dnmt1/PCNA/UHRF1 interactions can be associated with the Akt and/or PKC-mediated phosphorylation of Dnmt1, and that this disruption acts as an oncogenic event inducer of gliomagenesis by promoting the global DNA hypomethylation, which play an important part in chromosomal alteration and in the activation of proto-oncogene(s) by local hypomethylation (such as *PDGF-B, H-ras* and *MGMT* genes) (**Figure 9**).

## Discussion

Several papers demonstrate that PCNA and/or UHRF1 play a crucial role in the recruitment and/or the anchorage of Dnmts on DNA to maintain the DNA methylation pattern of mammalian cells *via* the capacity of these proteins to bind DNA and hemi-methylated DNA [6], [8], [12], [13], [14]. This work is the first or one of the first to identify the disruption of the Dnmt1, PCNA and UHRF1 interactions as a crucial oncogenic event promoting the DNA hypomethylation-induced tumorigenesis. Indeed, to date, the other model in which the DNA global hypomethylation promoted the tumorigenesis implicates the reduction of the Dnmt1 expression to 10% of wild-type levels in mice carrying a hypomorphic DNA methyltransferase 1 (Dnmt1) allele [10], [15]. Nevertheless, despite the central roles of the DNA methyltransferases in the establishment and the maintenance of epigenetic control, there was no evidence for the reduction of their expression as a significant contributing factor for cancer-associated hypomethylation [5], [16]. In our study, no Dnmt1 deficiency was observed in 9 primary cultured tumor cells and in 82 different glioma biopsies. Thus, the loss of Dnmt1 expression is not a frequent molecular determinant to the disruption of the Dnmt1-PCNA-UHRF1, contrary to the phosphorylation of Dnmt1 at serine-127 and/or at serine-143 by the Akt and PKC kinases. Indeed, mass spectrometry analysis, pull down assay and the synthesis of pDnmt1^S127^ and pDnmt1^S143^ antibodies validated the fact that these two kinases play a crucial role in the formation/disruption of the Dnmt1-PCNA-UHRF1 interactions and suggest that the use of these antibodies could be used as an alternative predictor of disease outcome since the high level of pDnmt1^S127^ and pDnmt1^S143^ in GBM seems to be associated with a poor prognosis factor of survival. Besides, this last point and the search of other mechanisms explaining the disruption of these interactions are two ongoing subjects of study in our lab (referred as “X event” in figure 9).

Moreover, the existence of (a) molecular mechanism(s) inhibiting the Dnmt1/PCNA/UHRF1 interaction is also supported by the paradoxical situation seen in astrocytoma cells. Indeed, the astrocytoma cells harbor a low level of Dnmt1/PCNA/UHRF1 interaction, whereas these cells, highly proliferative, are supposed to harbor a high level of the Dnmt1/PCNA/UHRF1 interaction according to the idea that the maintenance DNA methylation is a DNA replication-dependent process [17], [18]. In addition, this point participates to the debate to know the kinetic of the chromatin loading of Dnmt1 during the different phases of the cell cycle. Indeed, literature illustrates the debate by reporting, for example, that HDAC2 joins Dnmt1 and DMAP1 only during late S phase, that the association of Dnmt1 with the replication machinery enhances methylation efficiency, but is not strictly required for maintaining global methylation suggesting that the recruitment of Dnmt1 on DNA is DNA replication-independent, or against that the Dnmt1 is continuously loaded onto chromatin throughout the G2 and M phases [19], [20], [21], [22]. In our case, we noted that the reduction of cell in S phase (by a 10-fold factor) did not affect the number of Dnmt1/PCNA interaction (**Supplemental data S13**). More investigations are an ongoing in our lab and they need for taking into account the “available” of the various proteins taking part into the recruitment on the chromatin of Dnmt1 such as G9a, PCNA, UHRF1, HDAC1, or transcription factors such as E2F1 whose the expression and/or the post-translational modifications could be variable during the cell cycle [6], [7], [17], [23], [24].

To explain the tumorigenic mechanism associated with the genome hypomethylation induced by the disruption of the Dnmt1/PCNA/UHRF1 interactions, we demonstrate that the loss of these interactions is an event at the origin of the 1) chromosomal instability induced by the hypomethylation of DNA repeat element and 2) hypomethylation-mediated overexpression of specific genes such as the *PDGF-B, survivin, H-ras*, and *MGMT* genes i.e. genes coding for oncogenes or proteins participating to the acquisition of hallmarks of cancer [25], [26], [27], [28], [29]. Thus, these results clearly reinforce the causal link existing between the genome hypomethylation and the chromosomal instability because our results underline the presence of 9 deletions and 16 amplifications of chromosomal regions in hypomethylated Astro#40-UP cells. Interestingly, Astro#40UP cells are characterized by the deletion of the 18p11 and 14q32 chromosomal regions and by the 12q amplification, i.e. by chromosomal aberrations often observed in glioma [30], [31]. These results also support the idea that these regions contain oncogenes and/or tumor suppressor genes involved in the control of gliomagenesis.

By showing that the induction of global DNA hypometylation is an event able to induce the gliomagenesis from astrocytes (Astro#40 cells) and precursor of glial cells (Ntv-a cells), our data underline an oncogenic pathway common of mature and immature cells. Thus, the development of anti-glioma therapy targeting the limitation of the global DNA hypomethylation could be a successful therapy because glioma recurrence is frequently attributed to the resistance of stem cells or progenitors of glial cells.

## Materials and Methods

### ELISA

Microtiter plate was coated with capture antibody for overnight at 4°C. After 3 washes in PBS/Tween buffer (PBS pH 7.2–7.4, Tween-20, 0.05%), microtiter plate was blocked with 200 µl/well of blocking buffer (PBS pH 7.2, 10% Fetal calf serum) for 30 min at room temperature. After 3 washes in PBS/Tween buffer, samples are incubated for overnight at 4°C. After 3 washes in PBS/Tween buffer, detection antibody is incubated at the concentration of 2 µg/ml in 100 µl blocking buffer for 1 h at room temperature. Revelation is performed by incubating 50 µl/well of alkaline phosphatase conjugated secondary antibody diluted to 1∶500 in blocking buffer at room temperature for 1 h. Wells are then washed three times with PBS/Tween buffer and once with diethanolamine buffer (10 mM diethanolamine, 0.5 mM MgCl_2_ (pH 9.5) prior to pNPP substrate (Santa Cruz) addition in diethanolamine buffer to a final concentration of 1 mg/ml. Reaction is stopped by adding 0.1 M EDTA and read on microtiter plate reader at OD 405/490.

### Genetic and epigenetic analyses

DNA was extracted by using the QiaAmp DNA mini Kit (Qiagen, France).

Array-CGH experiments were performed by PartnerChip (Evry, France) using the Constitutional Chip 4.0 from Perkin Elmer.

Methylation array is performed by using the Promoter Methylation Array Kit according to the manufacturer's instructions (Ozyme, France).

Methyl-DNA ImmunoPrecipitation (MeDIP) and Methyl-DNA collection were performed by using the MeDIP kit™ and the MethylCollector Ultra kit according to the manufacturers's instructions (Diagenode, France and Active Motif, France, respectively).

Methyltransferase activities were estimated by performing DMB assay according to Yokochi and Robertson (2004).

### Olink/Duolink

Cells were fixed with 4% paraformaldehyde in PBS pH 7.4 for 15 min at room temperature. Permeabilization is performed with PBS containing 0.5% Triton 100× 4 for 20 min at room temperature and staining were realized according to manufacturer's instructions (Olink Bioscience). Fluorescence was visualized with ApoTome. 3D view was obtained by using Amira.4.1.1 program.

### Tumorogenicity assay

Cultured cells were harvested by trypsinization, washed and resuspended in saline buffer. Cell suspensions were injected s.c. as 10^6^ cells in 0.2 ml volume in the flank of 7/8-week-old Nude NMRI-nu female mice (Janvier, France).

### Preparation of Astro#40-UP cells

Astro#40 cells come from the Clonexpress Inc (Gaithersburg, USA). Ntv-a cells are a gift of Dr E.C. Holland. Astro#40-UP and Ntv-a/UP cells were obtained after nucleofection by using the Mouse Astrocyte Nucleofector™ kit (Amaxa biosystems, France) according to the manufacturer's instructions. Astro#40 cells were obtained from Clonexpress Inc (Gaithersburg, USA).

### Ethics statement

Human samples were collected according to French laws and the recommendations of the French National Committee of Ethics. The samples and the medical history of patients were encoded to protect patient confidentiality and used under protocols approved by the recommendations of the French National Committee of Ethics.

The experimental procedures using animals were in accordance with the guidelines of Institutional Animal Care and the French National Committee of Ethics.

### Supplemental experimental procedures

The Supplemental experimental procedures (**Supplemental data S14**) include lists of antibodies and primers used in all experiments and supplemental protocols.

## Supporting Information

Data S1 Characteristics of patients presenting low maintenance methyltransferase (mMTase) activity and High mMTase activity.
(0.11 MB TIF)

Data S2 Determination of the 5mC number in indicated cells by using flow cytometry method according to Hervouet et al. (Clin Cancer Res., 2009).
(0.03 MB TIF)

Data S3 Illustration of the control of the Dnmt1-immunoprecipitation performed by using the Catch and Release® v2.0 Reversible Immunoprecipitation System (Millipore, France).
(0.03 MB TIF)

Data S4 Effect of the farnesylthiosalicylic acid (FTS) (40 µM) treatment on the mMTase activity in Ntv-a/PDGF cells.
(0.03 MB TIF)

Data S5 SDS-PAGE and SyproRuby staining (in vitrogen, France) illustrating the fusion protein purification.
(0.05 MB TIF)

Data S6 Mass Spectrometry identification of PKC and AKT phosphorylation sites on rhDnmt1. Trypsin-induced rhDnmt1 peptides were obtained following to rhDnmt1 in vitro phosphorylation by PKC or Akt. Phosphopeptide enrichment was performed by IMAC and peptides were analysed by LC-MS/MS as described in Full Methods. MS spectra for the identified phosphopeptides are shown in Fig. S5a. Two phosphopeptides were identified in Akt-phosphorylated Dnmt1 (119–136 and 141–156) (lower spectra), one of which was also identified in PKC-phosphorylated Dnmt1 (119–136) (upper spectrum). Phosphopeptides were detected as 2× and 3× protonated peptides, as shown. CID (collision induced dissociation) MS/MS analysis was performed for both phosphopeptides on the 2× and 3× protonated species (MS/MS spectra recorded for the doubly charged ions at 931.0 m/z for the 119–136 peptide and at 874.9 m/z for the 141–156 peptide are shown in Fig. S5b). These spectra undoubtly revealed that only one serine residue is phosphorylated on each peptide, namely S127 and S143. The exact positioning of the phosphorylated serine could be evidenced by the observed loss of 69Da, a signature of a dehydrated serine after the loss of a phosphate group during the CID. In other terms, MS/MS analysis undoubtly revealed that only one serine residue is phosphorylated on each peptide, namely S127 and S143. Phosphopeptides were detected as 2× and 3× charged peptides, as shown for the first peptide. Of note, a minor peak corresponding to 2× charged peptide 119–136 could be detected in rh-Dnmt1. Nevertheless, it is obvious that this peptide, even if present in the control pool of tryptic peptides, is very minor since the intensity of the peak is comparable to that of non-phosphorylated peptides which were unspecifically bound by the IMAC. Besides, only the 2× charged peptide was detectable out of the background, contrary to the in-vitro phosphorylated conditions.
(0.19 MB TIF)

Data S7 Validation by ELISA method of the specificity of the pDnmt1^S127^ and pDnmt1^S143^ antibodies. Unphosphorylated recombinant Dnmt1 and Akt/PKC-mediated phosphorylated recombinant Dnmt1 were used to validate the pDnmt1^S127^ and pDnmt1^S143^ antibodies. The validation was confirmed by the fact that the pre-incubation of unphosphorylated peptides (P1-unP and P2-unP) not affected the detected of the pDnmt1, while the pre-incubation of the phosphorylated peptides (P1-P and P2-P) abrogated the detection of the pDnmt1. P1: RTPRRSKSDGEAKPEP and P2: MADANSPPKPLSKPRT phospho-serines are in bold.
(0.05 MB TIF)

Data S8 Validation by western blot analysis of antibodies directed against the pDnmt1^S127^ and pDnmt1^S143^. Recombinant Dnmt1 was phosphorylated as described in materials and methods section previous to be used as sample in western blot analysis.
(0.04 MB TIF)

Data S9 Expression of the pDnmt1^S127^ and pDnmt1^S143^ in PCTC using in figure 3A (A) and 3B (B). Ct-: negative control, Ct+: recombinant Dnmt1 phosphorylated by PKC or Akt.
(0.06 MB TIF)

Data S10 A: Position of primers and length of PCR products obtained after amplification of void vector or integrated UP insert. B: Detection of the integration of insert/constructs in vector and in Ntv-a cells using PCR. C: GFP expression after pCt or pUP nucleofection in Ntv-a. Similar data are obtained with the Astro#40 cells.
(0.13 MB TIF)

Data S11 Impact of the UP-induced disruption of the Dnmt1-PCNA-UHRF1 interactions on the methylation status of Alu by coupling the Methylated DNA COllection and PCR amplification (MeDCO) via the use of the MethylCollector Ultra kit (Active Motif, France). (I:input: M:Methylated and collected DNA).
(0.03 MB TIF)

Data S12 Expression level of the MGMT, PDGF-B, H-ras and survivin proteins in Ntv-a and Ntv-a/UP cells via western blot analysis.
(0.03 MB TIF)

Data S13 Cell cycle and Dnmt1-PCNA interaction. U251 cells were synchronised or not by serum starvation (72 h). Cell cycle phases were determined by using the NucleoCounter NC-3000TM Kit (Chemometec, France) and Dnmt1/PCNA interaction is determined by P-LISA method.
(0.04 MB TIF)

Data S14 Supplemental experimental procedures.
(0.06 MB DOC)
