# Supplementary figures and images for: Disruption of Dnmt1/PCNA/UHRF1 Interactions Promotes Tumorigenesis from Human and Mice Glial Cells
Source: PLoS One. 2010 Jun 29;5(6):e11333. doi: 10.1371/journal.pone.0011333 (PMC2894052; doi:10.1371/journal.pone.0011333)

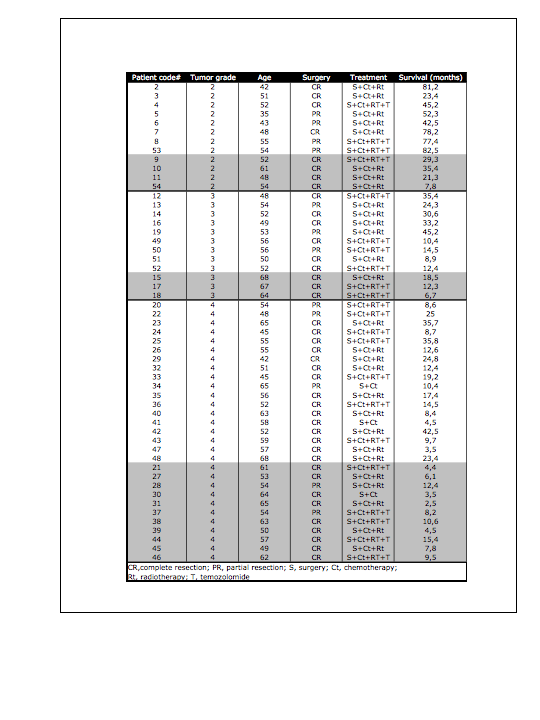

Supplement: Data S1 — Characteristics of patients presenting low maintenance methyltransferase (mMTase) activity and High mMTase activity. (0.11 MB TIF) [file pone.0011333.s001.tif]

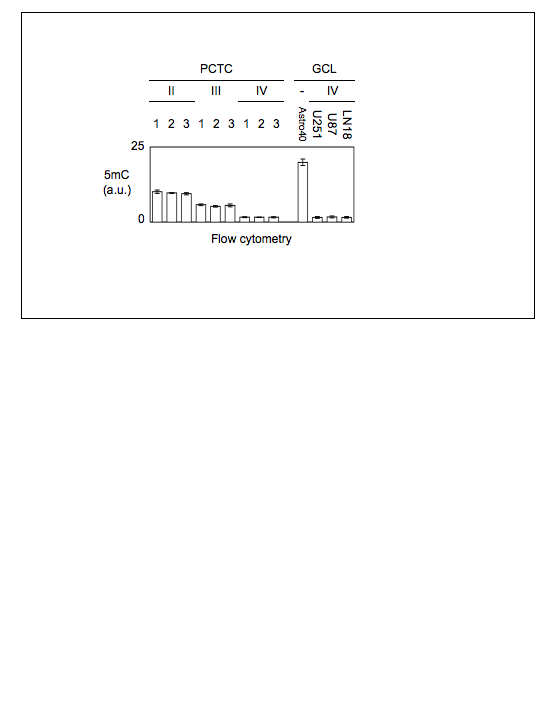

Supplement: Data S2 — Determination of the 5mC number in indicated cells by using flow cytometry method according to Hervouet et al. (Clin Cancer Res., 2009). (0.03 MB TIF) [file pone.0011333.s002.tif]

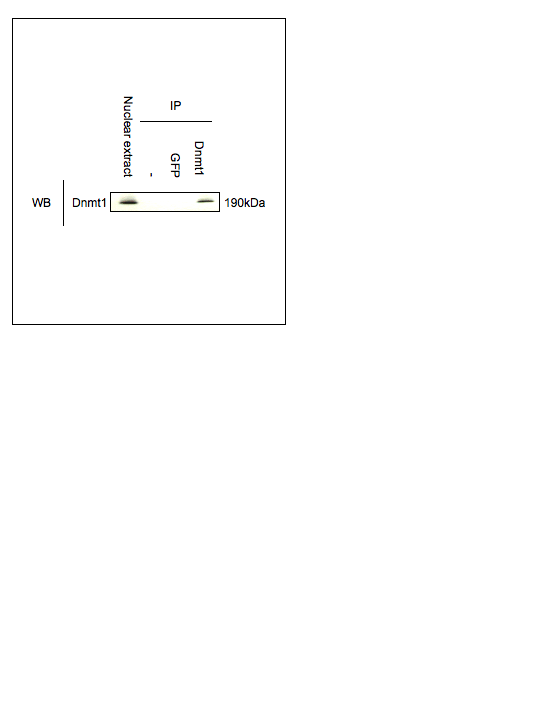

Supplement: Data S3 — Illustration of the control of the Dnmt1-immunoprecipitation performed by using the Catch and Release® v2.0 Reversible Immunoprecipitation System (Millipore, France). (0.03 MB TIF) [file pone.0011333.s003.tif]

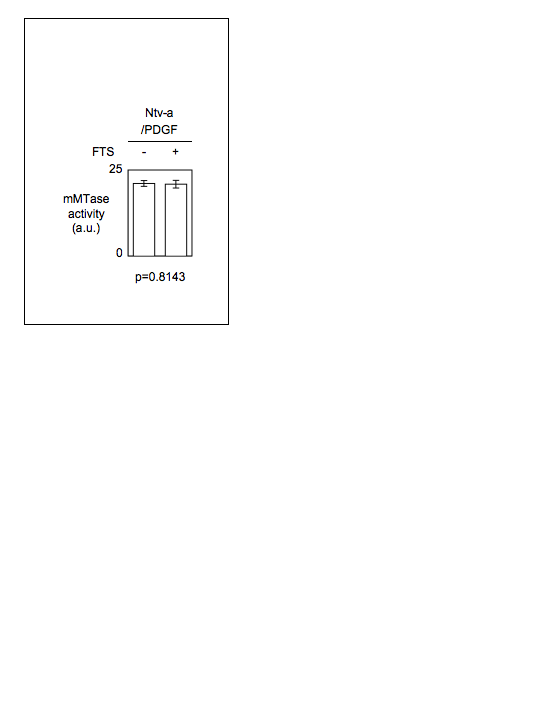

Supplement: Data S4 — Effect of the farnesylthiosalicylic acid (FTS) (40 µM) treatment on the mMTase activity in Ntv-a/PDGF cells. (0.03 MB TIF) [file pone.0011333.s004.tif]

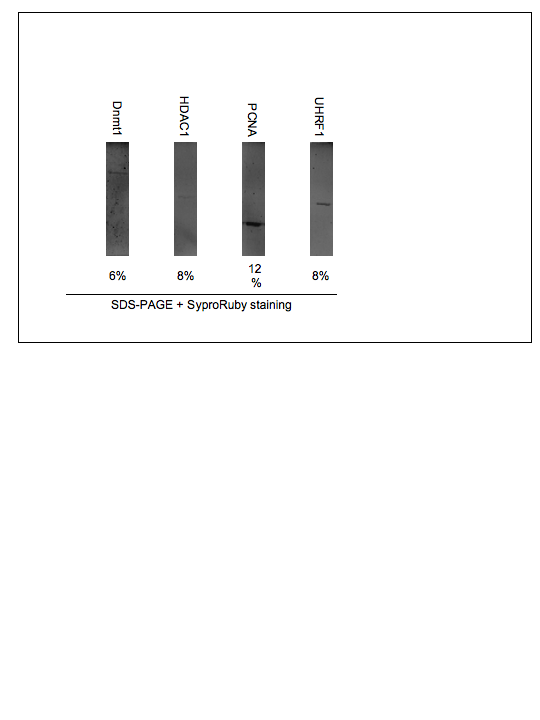

Supplement: Data S5 — SDS-PAGE and SyproRuby staining (in vitrogen, France) illustrating the fusion protein purification. (0.05 MB TIF) [file pone.0011333.s005.tif]

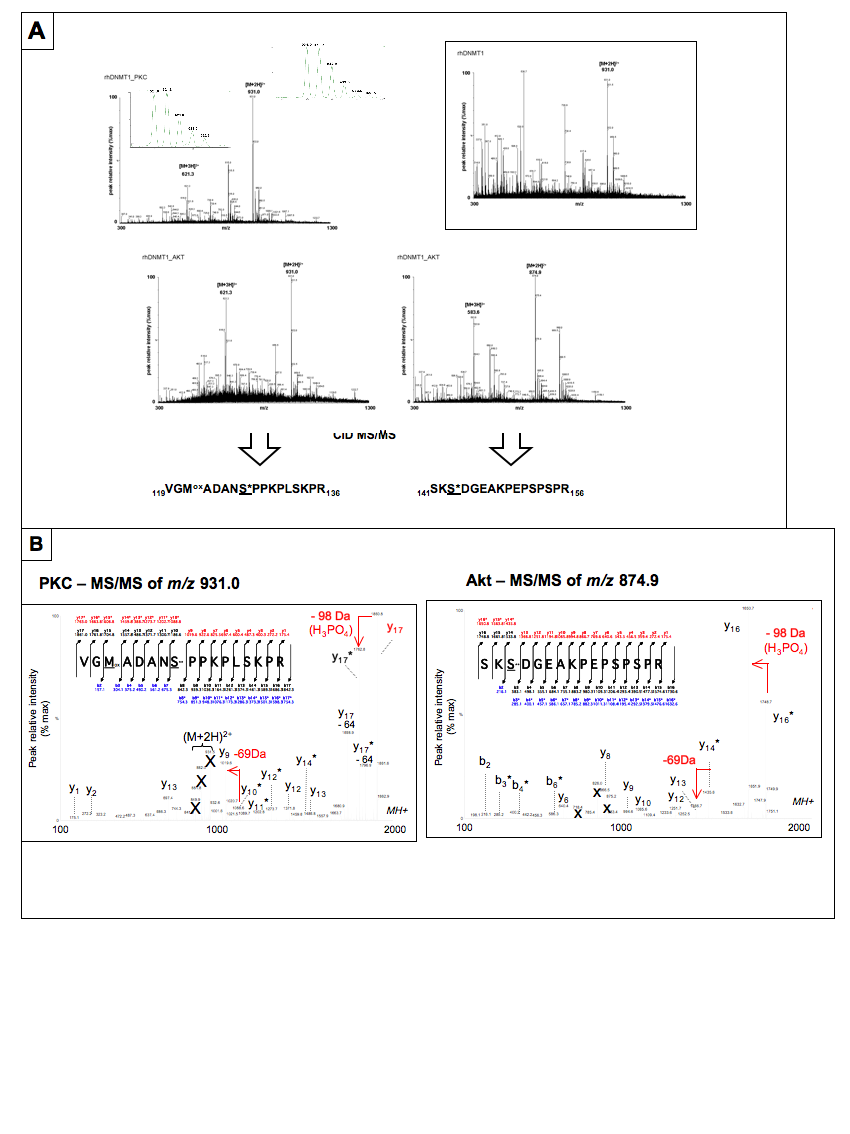

Supplement: Data S6 — Mass Spectrometry identification of PKC and AKT phosphorylation sites on rhDnmt1. Trypsin-induced rhDnmt1 peptides were obtained following to rhDnmt1 in vitro phosphorylation by PKC or Akt. Phosphopeptide enrichment was performed by IMAC and peptides were analysed by LC-MS/MS as described in Full Methods. MS spectra for the identified phosphopeptides are shown in Fig. S5a. Two phosphopeptides were identified in Akt-phosphorylated Dnmt1 (119–136 and 141–156) (lower spectra), one of which was also identified in PKC-phosphorylated Dnmt1 (119–136) (upper spectrum). Phosphopeptides were detected as 2× and 3× protonated peptides, as shown. CID (collision induced dissociation) MS/MS analysis was performed for both phosphopeptides on the 2× and 3× protonated species (MS/MS spectra recorded for the doubly charged ions at 931.0 m/z for the 119–136 peptide and at 874.9 m/z for the 141–156 peptide are shown in Fig. S5b). These spectra undoubtly revealed that only one serine residue is phosphorylated on each peptide, namely S127 and S143. The exact positioning of the phosphorylated serine could be evidenced by the observed loss of 69Da, a signature of a dehydrated serine after the loss of a phosphate group during the CID. In other terms, MS/MS analysis undoubtly revealed that only one serine residue is phosphorylated on each peptide, namely S127 and S143. Phosphopeptides were detected as 2× and 3× charged peptides, as shown for the first peptide. Of note, a minor peak corresponding to 2× charged peptide 119–136 could be detected in rh-Dnmt1. Nevertheless, it is obvious that this peptide, even if present in the control pool of tryptic peptides, is very minor since the intensity of the peak is comparable to that of non-phosphorylated peptides which were unspecifically bound by the IMAC. Besides, only the 2× charged peptide was detectable out of the background, contrary to the in-vitro phosphorylated conditions. (0.19 MB TIF) [file pone.0011333.s006.tif]

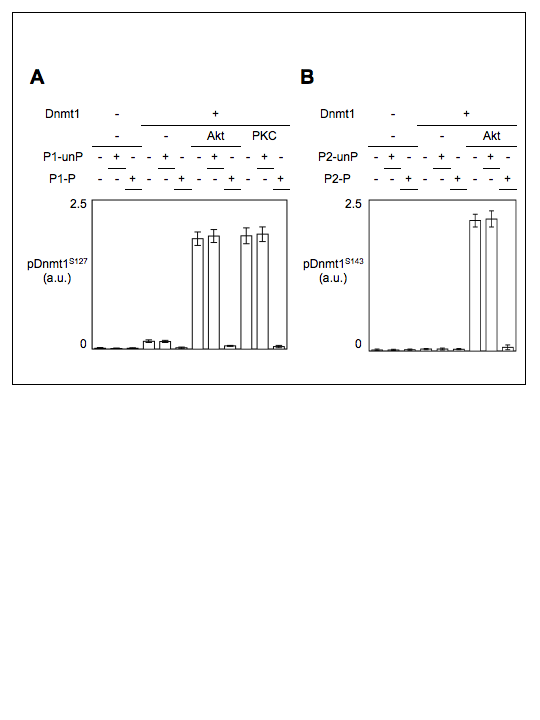

Supplement: Data S7 — Validation by ELISA method of the specificity of the pDnmt1S127 and pDnmt1S143 antibodies. Unphosphorylated recombinant Dnmt1 and Akt/PKC-mediated phosphorylated recombinant Dnmt1 were used to validate the pDnmt1S127 and pDnmt1S143 antibodies. The validation was confirmed by the fact that the pre-incubation of unphosphorylated peptides (P1-unP and P2-unP) not affected the detected of the pDnmt1, while the pre-incubation of the phosphorylated peptides (P1-P and P2-P) abrogated the detection of the pDnmt1. P1: RTPRRSKSDGEAKPEP and P2: MADANSPPKPLSKPRT phospho-serines are in bold. (0.05 MB TIF) [file pone.0011333.s007.tif]

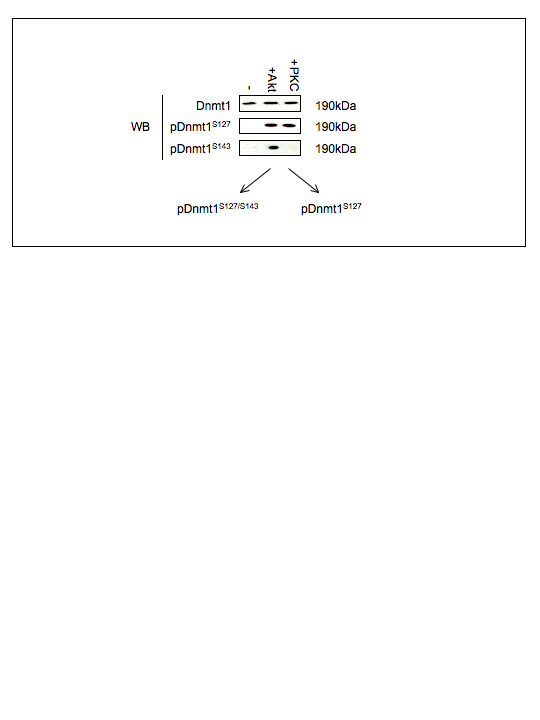

Supplement: Data S8 — Validation by western blot analysis of antibodies directed against the pDnmt1S127 and pDnmt1S143. Recombinant Dnmt1 was phosphorylated as described in materials and methods section previous to be used as sample in western blot analysis. (0.04 MB TIF) [file pone.0011333.s008.tif]

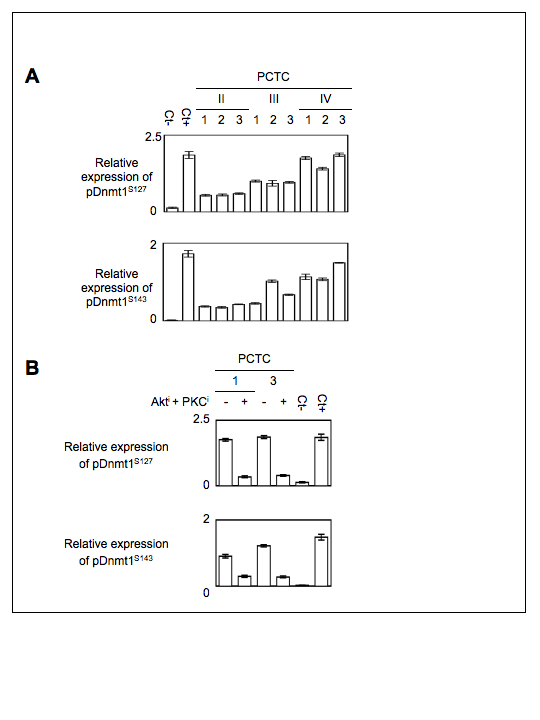

Supplement: Data S9 — Expression of the pDnmt1S127 and pDnmt1S143 in PCTC using in figure 3A (A) and 3B (B). Ct-: negative control, Ct+: recombinant Dnmt1 phosphorylated by PKC or Akt. (0.06 MB TIF) [file pone.0011333.s009.tif]

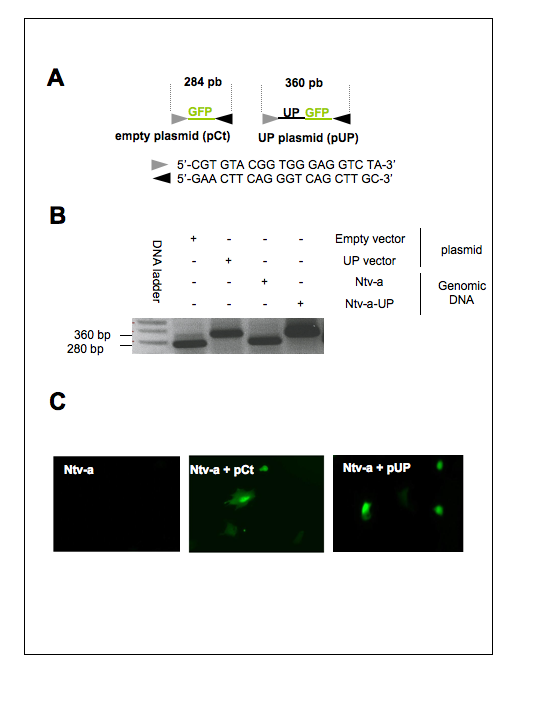

Supplement: Data S10 — A: Position of primers and length of PCR products obtained after amplification of void vector or integrated UP insert. B: Detection of the integration of insert/constructs in vector and in Ntv-a cells using PCR. C: GFP expression after pCt or pUP nucleofection in Ntv-a. Similar data are obtained with the Astro#40 cells. (0.13 MB TIF) [file pone.0011333.s010.tif]

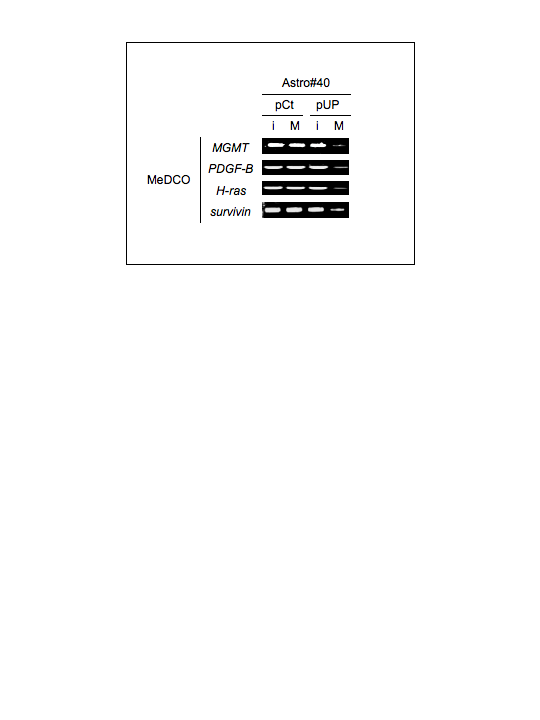

Supplement: Data S11 — Impact of the UP-induced disruption of the Dnmt1-PCNA-UHRF1 interactions on the methylation status of Alu by coupling the Methylated DNA COllection and PCR amplification (MeDCO) via the use of the MethylCollector Ultra kit (Active Motif, France). (I:input: M:Methylated and collected DNA). (0.03 MB TIF) [file pone.0011333.s011.tif]

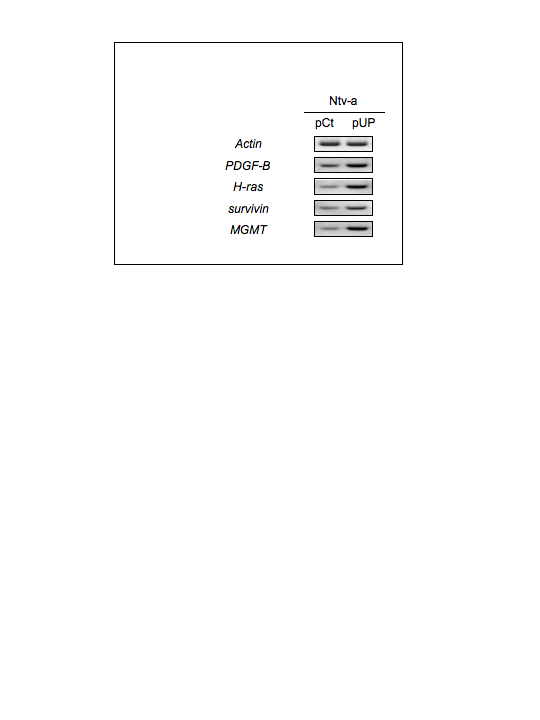

Supplement: Data S12 — Expression level of the MGMT, PDGF-B, H-ras and survivin proteins in Ntv-a and Ntv-a/UP cells via western blot analysis. (0.03 MB TIF) [file pone.0011333.s012.tif]

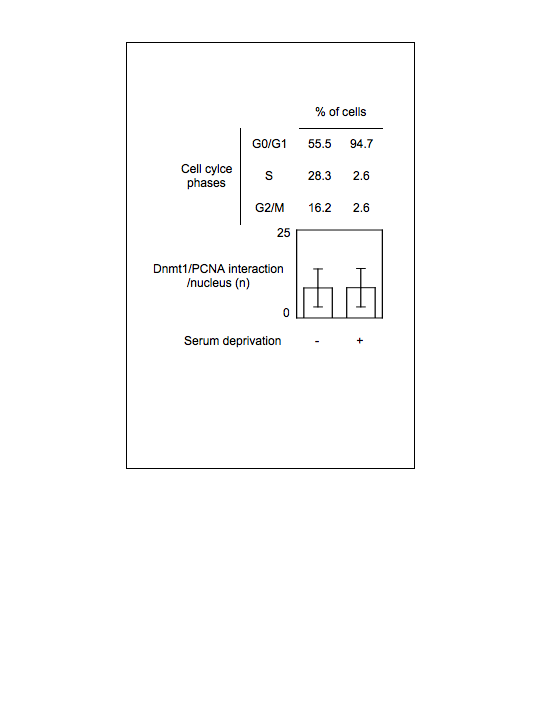

Supplement: Data S13 — Cell cycle and Dnmt1-PCNA interaction. U251 cells were synchronised or not by serum starvation (72 h). Cell cycle phases were determined by using the NucleoCounter NC-3000TM Kit (Chemometec, France) and Dnmt1/PCNA interaction is determined by P-LISA method. (0.04 MB TIF) [file pone.0011333.s013.tif]
