# Supplementary material for: Disruption of Dnmt1/PCNA/UHRF1 Interactions Promotes Tumorigenesis from Human and Mice Glial Cells
Source: PLoS One. 2010 Jun 29;5(6):e11333. doi: 10.1371/journal.pone.0011333 (PMC2894052; doi:10.1371/journal.pone.0011333)
Supplement: Data S14 — Supplemental experimental procedures. (0.06 MB DOC) [file pone.0011333.s014.doc]

**Supplemental experimental procedures.**

***Chromatin association experiments.***

Briefly, chromatin was purified from cells after crosslinking with 1% formaldehyde for 10 min at room temperature, 3 washes with PBS (pH 7.4) and lysis in buffer A (100 mM Tris-Cl at pH 7.5, 5 mM MgCl2, 60 mM KCl, 0.5 mM DTT, 125 mM NaCl, 300 mM sucrose, 1% NP-40). After lysis on ice for 10 min, the nuclei were pelleted, resuspended in buffer B (100 mM Tris-Cl at pH 7.5, 1 mM CaCl2, 60 mM KCl, 0.5 mM DTT, 125 mM NaCl, 300 mM sucrose), and added with 10 U of S7 nuclease for 20 min at 37 °C. The reaction was stopped by adding 50 mM EDTA. The chromatin was pelletted and resuspended in buffer C (1% SDS, 10 mM EDTA, 50 mM Tris-Cl at pH 8.0) overnight at 4 °C. After centrifugation (13000 rpm, 2min at room temperature), the supernatant was used for western blotting. All buffers contained PMSF and protease inhibitor cocktail (Sigma, France).

***Western blot.***

In brief, proteins were size fractionated by sodium dodecyl sulfate-polyacrylamide gel electrophoresis. Proteins were transferred onto nitrocellulose or PVDF membrane. Saturation and blotting were realized by using SNAP i.d™ Protein Detection System (Millipore, France). The detection of proteins was performed using ECL™(Amersham Biosciences) and/or SuperSignal west femto Maximum Sensitivity (Pierce) chemilumenscence reagents. Bands were quantified using Quantity One® quantification software (BioRad).

**Pull down Assay.**

Pull-down assays were performed by using the GST/His Tag Protein Interaction Pull-Down Kits (Thermo Scientic, France). Briefly, 100μg of bait protein is immobilized on column via an incubation at 4°C for 1 hour with gentle rocking motion on a rotating platform. After washes, 1μg of prey protein is added for 1 hour at 4°C with gentle rocking motion on a rotating platform. After washes and elution, the “bait-prey” interaction is analyzed by SDS-PAGE and western blot methods.

**Kinase assay.**

Kinase assay was performed in presence of 250ng of recombinant PKCα or Akt (Interchim, France) in 60 mM HEPES-NaOH, pH 7.5, 5 mM MgCl_2_, 5 mM MnCl_2_, 1.2 mM DTT, 100µM ATP, and the efficiency of these phosphorylation on the recombinant human His tagged Dnmt1 (rh-His-Dnmt1 - Methylation Ltd, Port Orange, Florida) was assessed by western blot and the used of the phospho(serine) PKC substrate antibody (PPCS) or the phospho(serine) Akt substrate antibody (PAS) (Cell Signaling/Ozyme. France)

**Identification of PKC- and Akt- phosphorylation sites on Dnmt1.**

*In vitro* phosphorylated Dnmt1 was separated by SDS-PAGE. Gels were fixed and stained with Sypro-Ruby according to the manufacturer’s instructions (Bio-Rad, France).

Protein bands were cut with surgical blades and subjected to in-gel proteolytic digestion as follows. Gels bands were washed with 25mM NH_4_HCO_3_, then with 50% acetonitrile in 25mM NH_4_HCO_3_. Proteins were reduced by 10mM DTT and alkylated by 55mM iodoacetamide (45min at room temperature). Gels bands were further washed with 25mM NH_4_HCO_3_ then with 50% acetonitrile in 25mM NH_4_HCO_3_ and proteins were digested overnight at 37°C by the addition of 300ng porcine trypsin (Promega, 15ng.μl^−1^ in 25mM NH_4_HCO_3_). The resulting peptide mixture was acidified by 2μl acetic acid 2.5M. Enrichment of phosphopeptides in the mixture was achieved by the use of IMAC spin columns (PhosphoProfile I, Sigma, France) according to the manufacturer’s instructions. Bound peptides were eluted by 2 x 25μl elution buffer containing 10% phosphoric acid, then dried in an air-vacuum centrifuge and stored at –20°C until analysis.

## **Mass spectrometric analysis**

Mass spectrometry analyses were conducted by the platform “Biopolymers-Interaction-Structural Biology” located at the INRA Center of Angers-Nantes (INRA UR1268, F-44300 Nantes) (<http://www.nantes.inra.fr/plateformes_et_plateaux_techniques/plateforme_bibs>).

Nanoscale capillary liquid chromatography-tandem mass spectrometry (LC-MS/MS) analyses of the digested proteins were performed using a Switchos-Ultimate II capillary LC system (Dionex, Amsterdam, The Netherlands), coupled to a hybrid quadrupole orthogonal acceleration time-of-flight mass spectrometer (Q-TOF Global, Waters, Manchester, UK). Chromatographic separation was conducted on a reverse-phase capillary column (Pepmap C18, 75μm i.d., 15cm length, Dionex) at a flow rate of 200nl.min^−1^. The elution gradient consisted of a linear increase from 2–40% acetonitrile over 50min, followed by a rapid increase to 50% acetonitrile over 10 min. Mass data acquisitions were performed with Mass software (Waters, Manchester, UK) using the ‘data dependent acquisition’ mode.

## **Protein identification – Databank searching**

LC-MS/MS data were processed using the Protein Lynx software (Waters) and further searched against databanks using Mascot 2.2 (Matrix Science). Protein identification was achieved by searching the peptide masses and MS/MS spectra against Uniprot/Swiss-Prot databank. One missed trypsin cleavage and two dynamic modifications (methionine oxidation and serine/threonine phosphorylation) were allowed per peptide. Mass accuracy was set to 150ppm for parent ions and 0.3Da for MS/MS fragments. Peptide sequences were validated with help of the OVNIp software (INRA Nantes, France. Tessier et al. *in preparation*). In both *in vitro* phosphorylated experiments, phosphorylated peptides were detected and MS/MS sequenced as doubly and triply protonated ions.

***List of antibodies.***

| **Antibody** | **References** |
| --- | --- |
| Actin | Chemicon#MAB1501R |
| PCNA | Abcam#Ab18197 |
| UHRF1 | Tebu#sc-57083 and Abcam#Ab-57083 |
| Histone H3 | Abcam#Ab1791 |
| Dnmt1 | Abcam#Ab13537 and Tebu#sc-10221 |
| Dnmt3a | Abcam#Ab13888 and Tebu#sc-20703 |
| Dnmt3b | Abcam#Ab13604 and Tebu#sc-20704 |
| PPCS | Cell Signaling#2261 |
| PAS | Cell Signaling#2324 |
| PDGF-B | Tebu#sc-7878 |
| H-ras | Tebu#sc-30 |
| MGMT | Lab vision#N77170 |
| survivin | Tebu#sc-8806 |
| Ki67 | BD Pharmingen#550609 |

***List of primers.***

| **genes** | **Localization** | **Primers** |
| --- | --- | --- |
| *PDGF-B* | -655/-553 | S:GCGCGCGGAGCAGCCGT AS:GGGCAGGGAGAGGTGCAA |
| *MGMT* | -557/-394 | S: ATTAGTGGAACACCCCAGCA  AS: TCCCGAAGACAAACTGTTCA |
| *H-ras* | -250/-113 | S:GAGCAAGTGGGGGCGAGG  AS: CTGCTGGCAGGGCCATCTGA |
| *Alu* |  | S: CATAGTTGGAACTCAGTCTCTC  AS: gggcatagtgaagtgatattg |
| *survivin* | -392/-241 | S: AGTAGAGACAAGGTTTCACCG  AS: GCCCCCTCGACTGCTTT |
